# Supplementary material for: The BRCAness Landscape of Cancer
Source: Cells. 2022 Dec 1;11(23):3877. doi: 10.3390/cells11233877 (PMC9738094; doi:10.3390/cells11233877)
Supplement: Supplementary file 1 [file cells-11-03877-s001.zip › Figure S1.pdf]

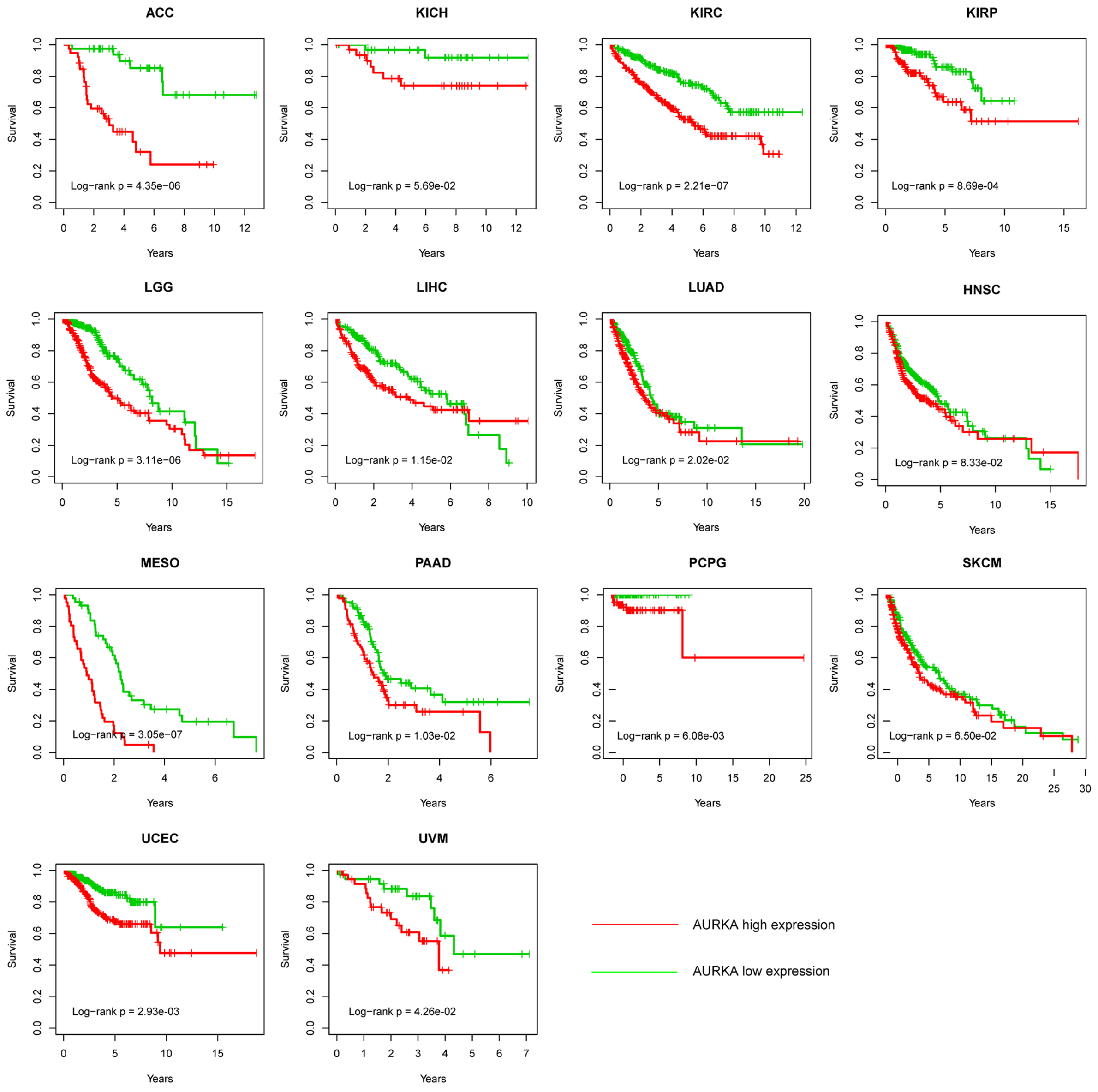

Figure S1. Kaplan-Meier survival plots of patients grouped by the expression levels of *AURKA* in 14 cancer types. P-values by log-rank test are shown in each cancer type.
